# Ingeborg Beling and the time memory in honeybees: almost one hundred years of research

**DOI:** 10.1007/s00359-024-01691-9

**Published:** 2024-03-12

**Authors:** Katharina Beer, Günther K. H. Zupanc, Charlotte Helfrich-Förster

**Affiliations:** 1 Behavioral Physiology and Sociobiology, Biocentre, University of Würzburg, Würzburg, Germany; 2 Laboratory of Neurobiology, Department of Biology, Northeastern University, Boston, MA 02115 USA; 3 Neurobiology and Genetics, Biocentre, University of Würzburg, Würzburg, Germany

**Keywords:** Time memory, Honeybees, Foraging, Circadian clock, Ingeborg Beling, Karl von Frisch

## Abstract

Bees are known for their ability to forage with high efficiency. One of their strategies to avoid unproductive foraging is to be at the food source at the right time of the day. Approximately one hundred years ago, researchers discovered that honeybees have a remarkable time memory, which they use for optimizing foraging. Ingeborg Beling was the first to examine this time memory experimentally. In her doctoral thesis, completed under the mentorship of Karl von Frisch in 1929, she systematically examined the capability of honeybees to remember specific times of the day at which they had been trained to appear at a feeding station. Beling was a pioneer in chronobiology, as she described the basic characteristics of the circadian clock on which the honeybee's time memory is based. Unfortunately, after a few years of extremely productive research, she ended her scientific career, probably due to family reasons or political pressure to reduce the number of women in the workforce. Here, we present a biographical sketch of Ingeborg Beling and review her research on the time memory of honeybees. Furthermore, we discuss the significance of her work, considering what is known about time memory today — nearly 100 years after she conducted her experiments.

## Introduction

Time-place learning is the process by which animals link events, such as finding food or potential mates, or encountering a predator, with both location and time of occurrence. The ability to remember the time and place of such events is useful because important events often occur regularly at a specific location. Thus, the association of the time and the place of an event allows animals to predict biologically relevant events. The capability to be at the right place at the right time appears to be particularly widespread among flying animals, such as birds, which arrive at a place from distant locations when resources are predictably available (Kamil 1978; Daan and Koene 1981; Rijnsdorp et al. 1981; Biebach et al. 1989; Krebs and Biebach 1989; Wilkie et al 1996; Tello-Ramos et al. 2015).

The first reports of time memory (German: *Zeitgedächtnis*) were, however, not related to birds but honeybees. About a hundred years ago, researchers began to investigate the remarkable time memory of honeybees. After first observations on honeybees that visited certain flowers at specific times of the day had been reported (von Buttel-Reepen 1990; Forel 1910), Ingeborg Beling was the first to examine this time memory experimentally and to establish its existence. In her PhD thesis, carried out under the supervision of Karl von Frisch,^1^ she systematically described the capability of honeybees to remember a specific time of the day, after they had been trained to a feeding place at restricted times. On the following day, she observed them returning—even when no food was provided (Beling 1929). In a set of behavioral experiments, she addressed the following three central questions: (1) How well are bees able to remember feeding times? (2) Is there a biological relevance to the time memory of bees? (3) Which factors (internal or external) influence the time memory of bees?

In this review, we will first present a biographical sketch of Ingeborg Beling. In the second part, we will review the early research on the time memory of honeybees, pioneered by Beling in her thesis and, a few years later, by Oskar Wahl, who also was a student of Karl von Frisch. In the third part of our article, we will discuss the significance of her work in light of what is known about time memory nearly 100 years after she conducted her experiments.

### Ingeborg Beling

#### The Munich years

Ingeborg Beling was born in Tübingen, Germany, on March 6, 1904.^2^ Her parents were Ernst Ludwig Beling and Toni Beling (née Helm). One year earlier, her father had joined the law school of the University of Tübingen.^3^ During 1912/13, he was rector^4^ of the university. Effective the summer semester of the latter year, he moved to the University of Munich, where he remained on the faculty until his death in 1932. In recognition of his contributions to criminal law, he was ennobled in 1912 and since then became also known as Ernst Ludwig von Beling, although he never used the aristocracy title.

In Munich, Ingeborg Beling attended the *realistische Abteilung des städtischen Mädchengymnasiums an der Luisenstrasse*,^5^ where she passed the *Abitur*^6^ exam in 1923.^7^ Between 1924 and 1929, she studied sciences — eight semesters at the University of Munich and two semesters at the University of Jena. Within these five years, she also completed her PhD thesis on time memory in honeybees.^8^ Her doctoral advisor was Karl von Frisch. As he mentioned in his evaluation of her thesis, the topic was inspired by reports in the literature and by *eigene Erfahrung* (his own experience). However, neither he nor Beling (1929) specified the kind of observations that had made him suggest the research project.

On the other hand, by Ingeborg Beling’s own account, we know more about von Frisch’s mentorship.^9^After she had told him about the first results of her experiments, he invited her to his family’s summer residence in Austria: “I have to see it myself—repeat the experiments in Brunnwinkl at Lake Wolfgang over the summer break.” During the weeks Beling spent in Brunnwinkl, she enjoyed not only his interest in her research and the enthusiasm he expressed when she reported new results, but also her warm welcome into his family. “In the wardrobe of my room, they even had put a Salzburg dirndl”—to match her outfit with those of his wife and his daughters. And, nearly five decades later, Beling also remembered the advice he gave her for writing up her thesis: “Read Gottfried Keller—this will improve your style of writing!”.^10^

**Fig. 1:**
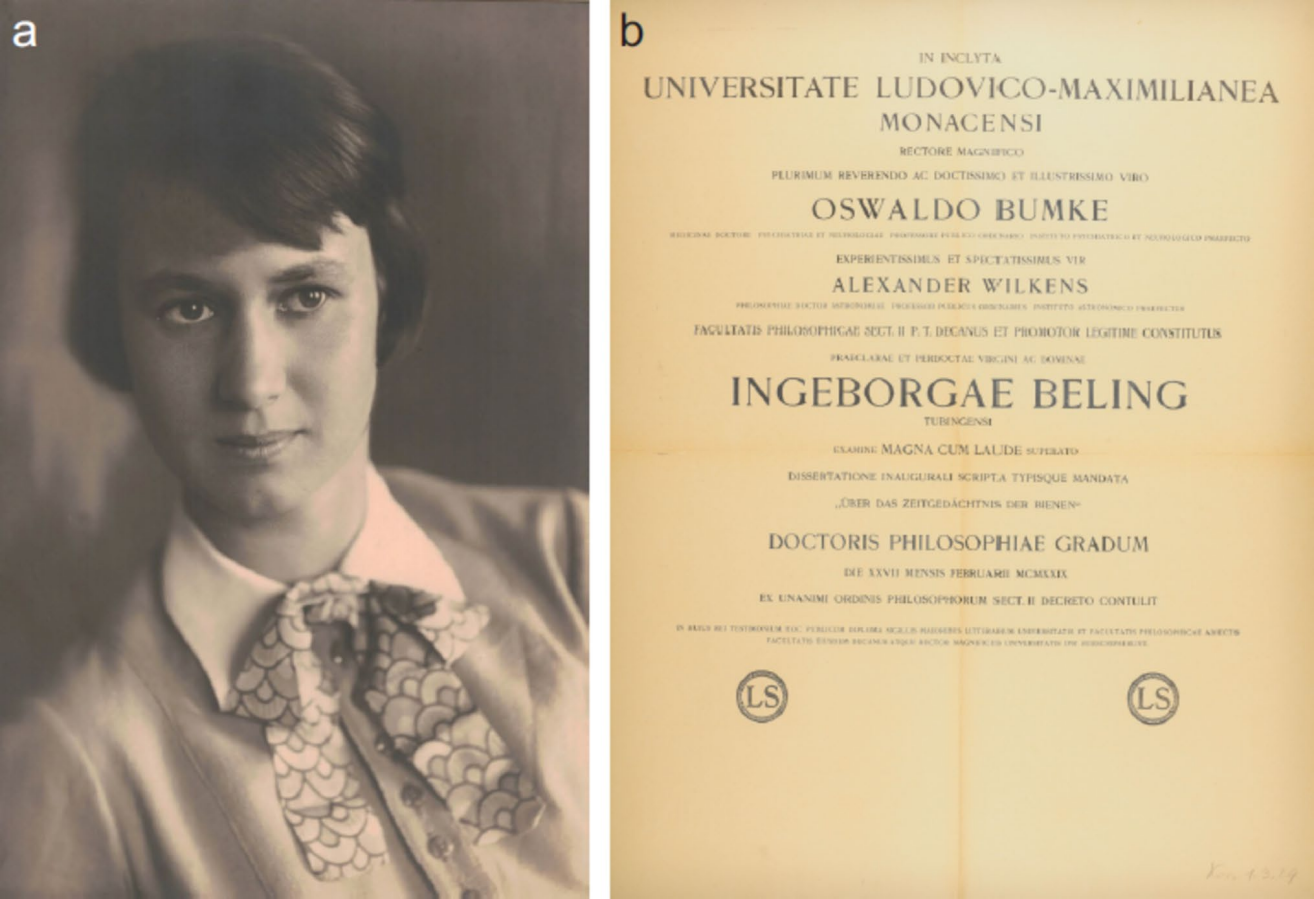
**a** Ingeborg Beling in 1929. Courtesy: Ingrid Bettin-Heitmann. **b** Ingeborg Beling’s PhD certificate. The document, written in Latin, lists, besides her name, the names of the rector of the University of Munich, Oswald Bumke, and the Dean of the Faculty of Philosophy, Sect. 2, Alexander Wilkens. It also shows the grade (magna cum laude), the title (in German) of her dissertation, *Über das Zeitgedächtnis der Bienen* (Time Memory in Honeybees), and the date of the award, February 27, 1929. Courtesy: Universitätsarchiv München (UAM OC-Npr-1928–29)

Ingeborg Beling successfully defended her thesis on February 27, 1929.^11^ Figure 1a shows a portrait photograph of her taken in the same year. For her thesis research, including the oral exams in her major, zoology, and her minors, botany and paleontology, she was awarded the second-highest honor, magna cum laude (Fig. 1b, right). Two papers resulted from this work—one published in the *Zeitschrift für vergleichende Physiologie* (Beling 1929), the other in *Naturwissenschaften* (Beling 1930a).

#### The Berlin years

Soon after receiving her PhD, in the summer of 1929, Ingeborg Beling interned for several weeks at the *Institut für Bienenkunde* (Institute for Apiology) in Berlin-Dahlem. Her project, suggested by the director of the institute, Ludwig Armbruster,^12^ involved the production of the first scientific film documenting how honeybees collect pollen from flowers.^13^ Since the quality of film shots in the field, with the then available technology, turned out to be unsatisfactory, Beling set up a hive and a ‘meadow’ made of freshly cut flowers in a ‘constant room’ (in which temperature and level of illumination by artificial light were kept constant)—like the room she had used for her thesis research (Beling 1929). This arrangement facilitated close-up shots and slow-motion pictures for analysis of the bee’s rapid body movements during pollen collection. Beling published the results of this analysis, including some of the key frames of the film, in an accompanying paper (Beling 1931a).

A year later, on September 15, 1930, Ingeborg Beling commenced a position at the *Biologische Reichsanstalt für Land- und Forstwirtschaft* (Biological Reich Institute for Agriculture and Forestry), which, like the *Institut für Bienenkunde*, was in Berlin-Dahlem.^14^ The fact that, as a woman, she had managed to get employed in her field after completion of her academic education is worth mentioning. Whereas female students were not uncommon any more at universities in the Weimar Republic by that time (between 1930 and 1933, nearly one out of five students was a woman^15^), women were severely underrepresented among staff scientists and virtually non-existent among senior ranks, including professors. This discrepancy was also evident at the *Biologische Reichsanstalt.* On March 1, 1933, Beling was listed as the only woman among 64 scientists.

At the *Biologische Reichsanstalt*, Beling worked in the Laboratory of Physiological Zoology headed by Albrecht Hase.^16^ What seems to have been her first project was a study commissioned by I. G. Farbenindustrie AG, a major German chemical and pharmaceutical conglomerate. They had asked Hase to examine the moth-proofing effect of their product ‘Eulan WA new.’ Beling, who conducted the experiments, showed that this chemical compound (its active ingredient is chlorphenylid), indeed, renders samples of wool proof against damage by the larvae of the common clothes moth (*Tineola bisselliella*) (Beling 1930b). Over the following decades, Eulan WA new was widely sold for protecting textiles and other materials against moth damage.

During the next few years, her research mainly focused on exploration of the potential use of parasites for biological pest control of the Mediterranean flour moth (*Ephestia kuehniella*). The larvae of this moth feed on a variety of foods, including wheat flour, grains, cereals, dried fruits, and nuts. They generate an unpleasant odor and leave frass that contaminates the food stuff. At the same time, they produce webbing that may clog and damage machines in the mill.

One of the parasites that Beling studied was the ichneumon wasp *Venturia* (= *Nemeritis*) *canescens*. By breeding this endoparasitoid wasp over several generations, she succeeded in a comprehensive description of its parthenogenetic reproduction, its development from eggs deposited inside the larvae of the moth, and the feeding behavior and orientation of the adult wasps outside the mill (Beling 1932a; von Stein-Beling 1934). Along a similar line of research, she reported for the first time the ichneumon *Angitia armillata* as an endoparasite of larvae of *Ephestia kuehniella* (Beling 1933).

It is unknown for how long Ingeborg Beling remained on the scientific staff of the *Biologische Reichsanstalt*, but her personal notes indicate that in 1937, she was no longer employed by this institution. Her last scientific article appeared in 1935, marking the eleventh paper published within seven years (Beling 1929; Beling 1930a, b, c, 1931a, b, 1932a, 1933; b; von Stein-Beling 1934, 1935). This was a remarkable publication output, placing her par with some of the most productive German zoologists of her time. We also do not know the reason for the very early retirement from her position at the *Biologische Reichsanstalt*. However, it is likely that it was related to her marriage (to Rudolf Freiherr von Stein zu Nord- und Ostheim^17^) in 1932; the birth of the first of her three children in 1934^18^; and/or Nazi policies that combated *Doppelverdienertum* (double-earning households) by reducing the number of married women in the workforce.^19^

#### The later years

A letter written by Karl von Frisch to Ingeborg von Stein-Beling on March 20, 1952, indicates that he had learned that, together with her daughter, she had moved away from the family residence in Heigenbrücken (a municipality in the Aschaffenburg district in Lower Franconia, Bavaria), where her husband worked as a forester. Evidently, von Frisch saw this as an opportunity to recruit her.

*You would do us and science a great service if, as an assistant at the Zoological Institute, you were willing to carry out research on bees, commencing your position on May 1*^*st*^*. I have received funds from the Rockefeller Foundation for this work so that I can hire you. Your expertise based on your previous work would be invaluable. I very much hope that you will accept my invitation.*^20^

Although our search in the Archives of the University of Munich failed to find any documents that would indicate employment of von Stein-Beling at any time after she had received her PhD in 1929, her daughter remembers it being around that time her mother rejoined the Zoological Institute headed by von Frisch.^21^ This is also mentioned in an essay that Ingeborg von Stein-Beling dedicated to Karl von Frisch on the occasion of his 90th birthday in 1976.

*25 years later [after the dissertation work] I had the honor to work once again, for a short time, under his [Karl von Frisch’s] supervision in a team at the new Munich institute…*^22^

Ingrid Bettin-Heitmann furthermore remembers that, presumably in the summer of 1952, Karl von Frisch took her and her mother in his car from Munich to Brunnwinkl, where film shots were made while they worked with bees. She later recognized her mother as an assistant in the scientific film *Die Tänze der Bienen* (The Dances of Bees)*.*^23^ Part of this film, including scenes with Ingeborg von Stein-Beling, can be seen in the 2013 documentary ‘More than Honey’ directed by Markus Imhoof.^24^

The specific topic on which Ingeborg von Stein-Beling worked at the Zoological Institute of the University of Munich is unknown. Searches in various databases, such as PubMed and Web of Science, have not revealed any publication (co-)authored by her after 1935. It also remains elusive why she left the institute after a rather short stint. Officially, she moved to Kleinwallstadt in the Miltenberg district of Lower Franconia on December 5, 1952, where her husband had assumed a new position as forester. She maintained a legal residence there until she passed away in Berlin-Steglitz on January 15, 1988.^25^

### Early investigations on the time memory of honeybees

#### Experiments by Ingeborg Beling and Oskar Wahl

In the following, we will describe Ingeborg Beling’s thesis research on the time memory of honeybees. This work appeared in German in the *Zeitschrift für vergleichende Physiologie*, the predecessor of the Journal of Comparative Physiology A (Beling 1929). A few years later, Oskar Wahl^26^ expanded Beling’s research and published his study in the same journal (Wahl 1932).

Beling (1929) offered honeybees food at an outdoor feeding station, which she gradually set up to ensure that the bees visited the artificial food resource. Initially, bees were attracted with honey offered in the immediate vicinity of the hive, and the arriving bees were marked. Then, the honey was replaced with sugar water (50%) (and, in one experiment, additionally with pollen powder), and the feeding station moved stepwise to a foraging distance. The feeding station usually consisted of a food container put with its open side down on blotting paper on a table. This setup enabled many bees to lick sugar water off the blotting paper simultaneously. Furthermore, the sugar water supply lasted for hours, so the experiments did not have to be disrupted by refilling the container.

In the constant-room experiments, Beling trained the bees like in the field experiments. However, they were confined to foraging only in an approximately 90 m^3^ room that had no windows and was painted black. Moreover, its temperature and humidity were kept stable. A field hive was moved before the start of the experiments into this constant room and placed on a 1.4 m long table, together with a food source, which was 30 cm from the hive. Constant-light conditions were provided by an incandescent lamp. After two days of feeding, the bees were accustomed to visiting the feeding station. Beling then began the training of the bees, offering food during time windows of 1.5–4 h. The training session usually lasted for a few days but was extended to as long as 3 weeks if the weather was rainy. The training was followed by a test day of continuous observations at the feeding site, without offering food. All visiting bees were recorded, including those that came to the feeding site more than once (as long as they had been away from the feeding site for at least 1 min).

The results showed that it was possible to train the bees to visit the feeder at certain times of the day (Beling 1929). At all other times, bees rarely visited the feeder. Most significantly, foraging activity at the feeder increased shortly before the time of day when the bees were used to find food at the feeder, peaked immediately before or during the expected feeding time window, and stopped shortly thereafter.

**Fig. 2:**
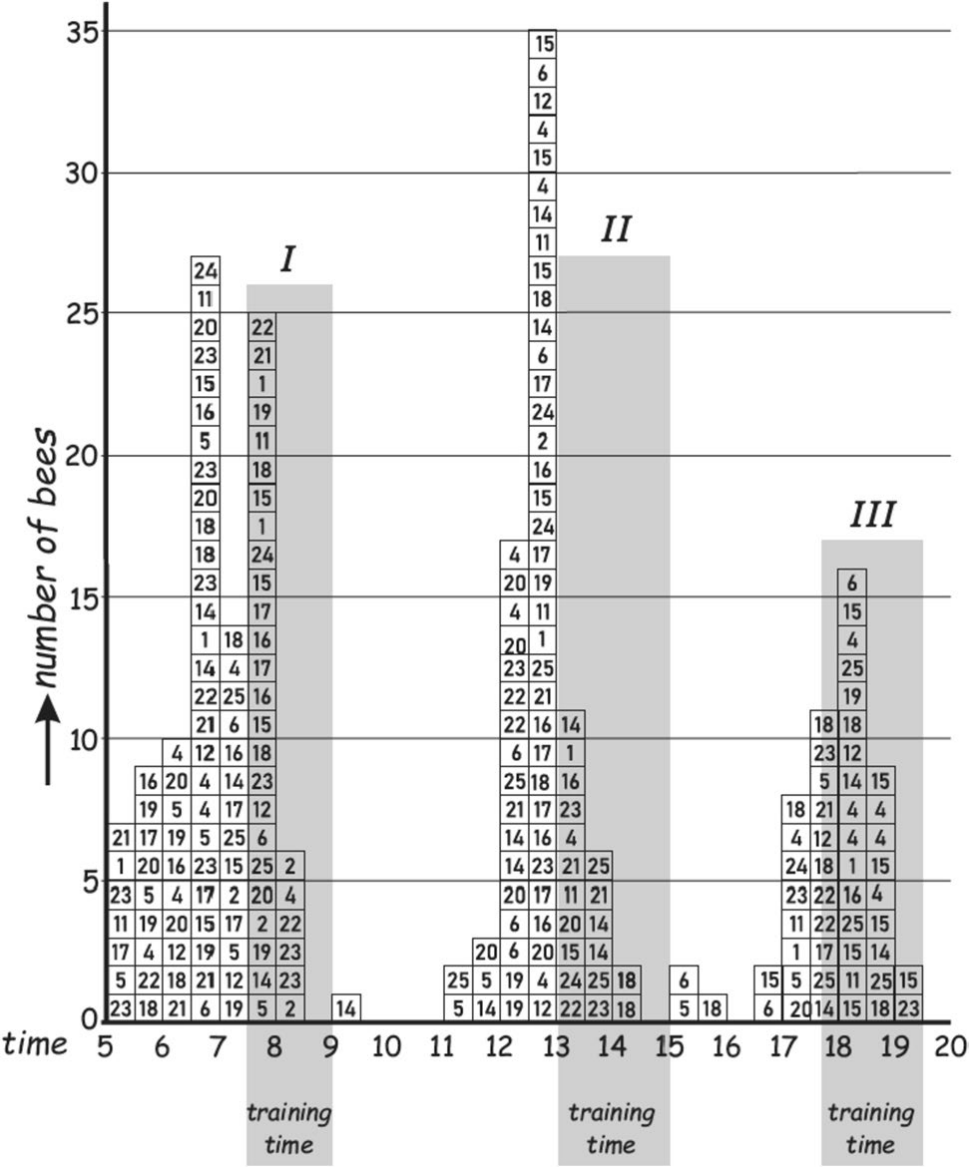
Table-figure showing time memory after training field bees at 3 daytimes. Training times are shaded in the figure: 7:30–9:00, 13:00–15:00, 17:45–19:30. Six training days preceded the test day. Out of 19 marked bees, 19 visited the feeder on the observation day, without food presented. Individuals are listed with marked numbers. Bees are counted in time bins of 30 min in daytime hours 5 to 20. (Adapted from Beling 1929.)

Beling tried different feeding times, as well as several feeding times during the day (Fig. 2). In all cases, the bees were able to remember the feeding times they were trained to. It was also possible to retrain them to other time windows. These observations led Beling to conclude that the time memory of honeybees is highly plastic, while it enables them to precisely recall one or several times of the day when food is available.

In the constant-room experiments with artificial light, Beling also found that the time memory is not limited to the daytime. Rather, bees can remember the time of feeding at any daytime or nighttime. Remarkably, bees could remember feeding times even after just one day of training.

Shortly after Beling’s thesis research, Oskar Wahl (1932) investigated further the bees’ ability to remember feeding times. By offering food continuously and checking whether bees visited the feeder place at preferred times of the day, he added an important control experiment to Beling’s studies. He found that untrained bees had no preferred feeding times, which supported Beling’s conclusions. He also confirmed Beling’s findings that bees can be trained for several feeding times. Furthermore, he determined the minimum interval between two training times necessary for the bees to memorize both; it was at least 2 h. With a day length of approximately 16 h on the longest day of the year in Munich, this indicates a memory capacity of 5–6 different feeding times per day. (Oskar Wahl did not consider longer days at higher latitudes or constant light in this calculation.) Furthermore, he confirmed Beling’s conclusion regarding a highly plastic time memory in bees by training them to a novel time point. They preferred this new time after just two days of training. If no food was offered at any other time, and no other food sources were available (in the constant room or in late season with few natural resources available), the bees’ time memory lasted for at least 6 days after removing the food from the feeder (Wahl 1932).

#### Adaptive advantage of the bee’s time memory

It was obvious to Beling (1929) that the honeybee’s extraordinary capability to remember feeding times, and the remarkable plasticity in rapid reprogramming the memory to other feeding times, must have evolutionary relevance. She writes that the time memory is probably an adaptation to biological properties of flowers. Since several flowers open and close at certain times of the day, as Linnaeus (1751) showed in his Flower Clock,^27^ the bees can be ‘trained’ to these times by the plants. Remembering the times when the flowers are open and offer nectar and pollen optimizes foraging. Indeed, von Buttel-Reepen (1990) observed that bees visit the flowers of buckwheat only in the morning hours until approximately 10 am, which coincides with the time of its nectar production (Beutler 1930). Dolgova (1928) investigated the proposed coevolution between flowers and bees in more detail and established daily curves of bee visitation activity, with defined hours of maximum visitation for several bee food plants. While this is a good indication of what bees use their temporal memory for in nature, Beling (1929) argued that more research is needed to determine whether nectar production or simply access to open flowers is the primary cause of the cyclical foraging.

On a side note, Beling (1929) addressed the question of whether the bees’ time memory serves to simultaneously remember the location of a food source and the time when food is available at this location. Wahl took up this question and trained bees to two different locations, each with a different feeding time. In the test run, the bees searched more intensively for food at the trained location associated with the correct time (Wahl 1932). Between the feeding times, the bees did not visit either feeding place. This indicates that bees are able to exhibit time-place learning, which allows them to visit flowers at different places at corresponding different times, thereby exhibiting a highly efficient foraging strategy. In other words, the bees would waste time and energy if they visited flower patches that were not rewarding at the time of the bee’s foraging.

However, the questions remained of how bees orientate, whether time memory is interconnected with place memory, and how bees form time-place-memory. Beling assumed that the sun’s movements on the sky serve them as orientation cues. However, she argued that there must be other mechanisms underlying orientation because the bees can also recall the time correctly when the sun is not visible. These questions were only answered several years later by Karl von Frisch, Martin Lindauer, and their groups (see below).

#### The clock underlying the time memory

When Beling carried out her experiments, the concept of a circadian clock was not yet known, although it had previously been shown that plants exhibit rhythms in leaf movements that are independent of the daily light–dark cycles and could therefore be based on an endogenous clock (Duhamel Du Monceau 1758; de Candolle 1832; Pfeffer 1915; Stoppel 1926). Unfortunately, this work was not well known, and the results were not accepted by the scientific community when Beling did her thesis research.

It was one of Beling’s goals to study the underlying clock that helps the honeybee to measure time. She excluded the possibility of an absolute sense of time in honeybees and assumed that bees measure the time interval between feeding times. Since Beling was working with feeding cycles, she first investigated whether hunger drives such cycles. She ruled out this hypothesis, arguing that bees can be trained to feed at several times a day. It is highly unlikely that the bee will eat just the right amount each time, only to become hungry again at the next feeding time window. Furthermore, this mechanism would only help to determine the beginning of the feeding window, but not the end. However, in experiments bees were able to clearly distinguish between feeding intervals of different lengths. Further evidence supporting the notion that hunger is not the driving force behind the trained feeding rhythms was: first, Beling could not determine any specific feeding times of the bees in the hive on rainy days; second, she found food in the bees’ midgut at any time of day (Beling 1929). Wahl confirmed this result by systematically tracking specific individuals that practiced trophallaxis at all times of the day (Wahl 1932). It, therefore, seemed highly unlikely that the bees needed to be hungry when they flew to the feeding site.

Another interesting observation was that it was impossible to train the bees to any cycle other than 24 h. Beling fed the bees for 16 days every 19 h, but the bees were not able to learn this feeding cycle. Only after the last feeding, she observed a small maximum of food searches after 24 h but not after 19 h (Beling 1929). Consistent with these findings, Wahl was unable to train bees to a feeding interval of 48 h. The bees appeared at the feeding sites every 24 h, which led him to conclude that time memory is inextricably linked to the 24 h cycle (Wahl 1932).

Beling (1929) reasoned that the ‘clock’ by which the honeybee memorizes feeding times can be based either on external (environmental) factors or on an internal process with a periodicity of 24 h (internal clock). Initially, she suggested some kind of unknown cyclic signal from the brood as a timer for the bees. She quickly ruled this out, as colonies in the constant room without brood were just as trainable to a feeding time as colonies with brood. Within the same context, she demonstrated that bees born in an incubator without ever having experienced life in the wild could be trained to a feeding time just as well as bees born in a hive. In a more systematic way, Wahl raised an entire hive, including the queen, in an incubator and then successfully tested the trainability of feeding rhythms (Wahl 1932). Both Beling and Wahl concluded that the clock used by the bees for time training is inherited and not dependent on cyclic signals from the brood.

Next, Beling systematically excluded the daily changes in light, temperature, and humidity by keeping these environmental factors constant in her constant-room experiments. She also excluded the daily changes in the electrical conductivity of the air by disturbing these environmental fluctuations through irradiation of the experimental chamber with a radioactive source. Under any of these conditions, the bees showed the usual ability and pattern of returning to the foraging sites after daily training. Again, Wahl built on these results by conducting an experiment in which he measured time memory in a salt mine, which almost no cosmic radiation can reach (Wahl 1932). The underlying rationale was that, if there is no other, unknown oscillating environmental factor on Earth, the honeybee most likely uses an internal clock to measure time.^28^

**Fig. 3:**
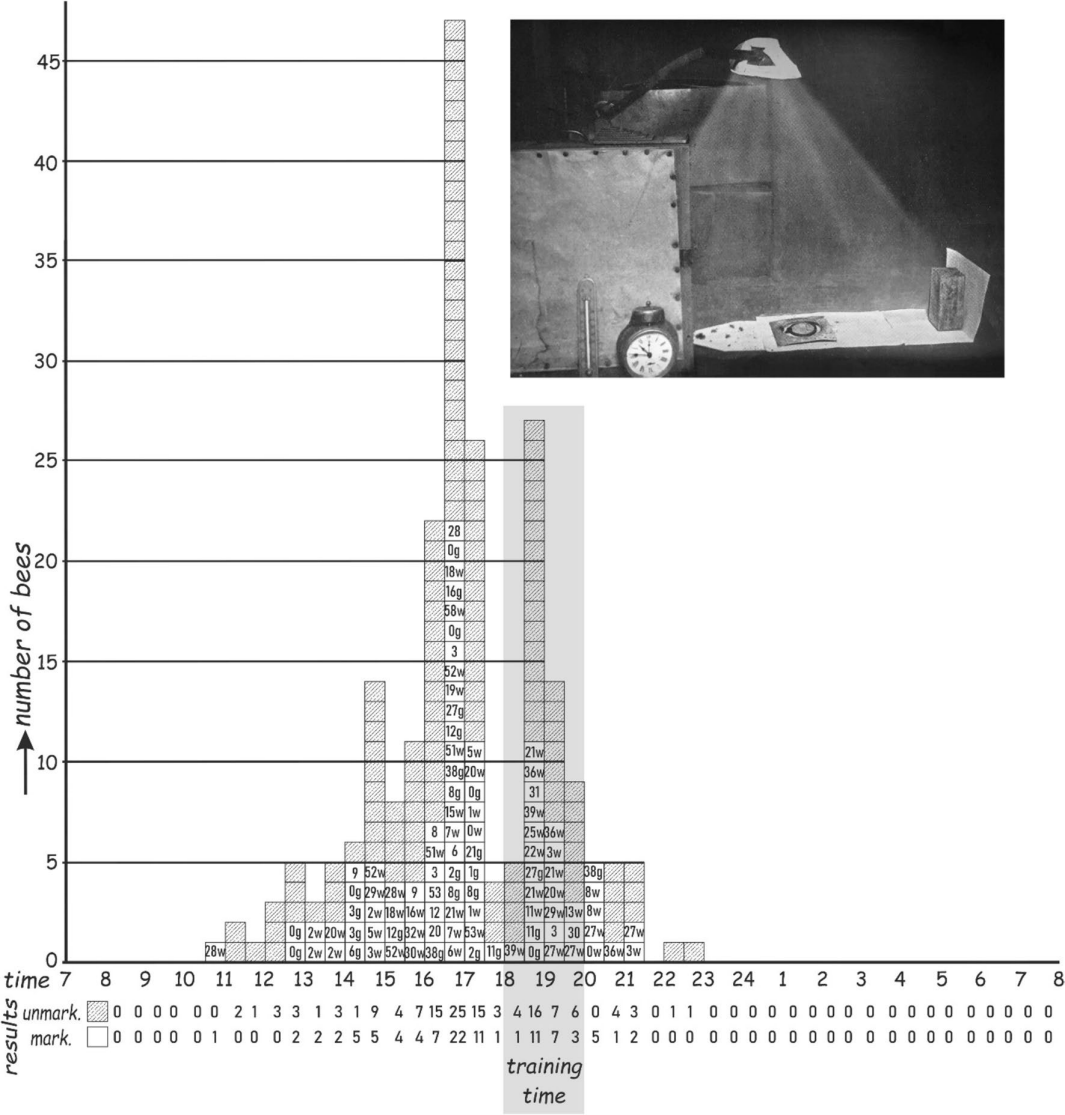
Table-figure demonstrating appearance of the bees at the feeder clearly before the trained time in a constant room experiment with low constant light (see inset). Training time is shaded in the figure: 18:00–20:00. Training took place over 12 days. 45 out of 50 marked bees (individuals with numbers) and additional unmarked bees (gray squares) visited the feeder on the observation day without food present. ‘w’ = white,‘g’ = yellow marking colors. Counts of ‘marked’ or ‘unmarked’ bees are beneath the time scale (time bins of 30 min in a full 24 h cycle starting at 8 am). (Adapted from Beling 1929.)

Under constant light in her experimental chamber, Beling (1929) observed that the peak of visits by bees was always earlier than the peak of visits in field experiments (Fig. 3). She did not consider this to be a methodological error because the same bees were able to be trained to any time point during the day. Thus, one must assume that the clock underlying foraging runs faster under constant laboratory light conditions than under natural conditions—a finding that was later confirmed (Frisch and Aschoff 1987). Wahl (1932) added to these observations a very interesting experiment in which he compared the timed foraging of bees at two different temperatures (approx. 23 °C vs. 31 °C) in a constant room and hive (the colony was without brood and did, therefore, not thermoregulate). When he compared the two foraging curves of the trained bees, he could not find a difference and concluded that the factor that influences the time memory regulation cannot be a mechanism based on metabolism, as the rhythm should accelerate with the metabolic activity at higher temperatures. This strongly reminds us of Bünning’s observations on the rhythmic leaf movements of *Phaseolus* bean plants, which showed only a slight acceleration at higher temperatures compared to other metabolic processes (Bünning 1931). In 1954, Pittendrigh formulated the concept of temperature compensation of circadian rhythms, which became an important feature in chronobiology (Pittendrigh 1954).

In their work on the time mechanism used by foraging honeybees, Beling (1929) and Wahl (1932) described the following basic characteristics of the circadian clock (although they did not call the clock as such):

1. the rhythm continues under constant conditions (e.g., in a salt mine);
2. it appears to free-run with a period that slightly deviates from 24 h (under constant conditions, the bees appeared earlier at the feeding place, which suggests a free-running period of < 24 h);
3. it has a limited entrainment range, as it cannot follow any training cycle (19 h and 48 h are not possible);
4. it appears to be temperature compensated because its period does not deviate from approximately 24 h at 23 °C and 31 °C.

In summary, the work of Beling and Wahl can be regarded as a historical milestone, providing the first convincing evidence that circadian systems permit organisms to measure time for adaptively significant purposes, namely to forage only when nectar and pollen are available. Later work on the time memory confirmed the findings of Beling and Wahl.

### Investigations on the time memory of honeybees after Beling and Wahl

#### Coevolution of plant–bee interactions

In 1933, Wahl showed that honeybees can also remember the time of day at which nectar secretion by a certain flower is maximal or contains the highest amount of sugar (Wahl 1933). This ability is highly adaptive because several flowers secrete nectar rhythmically (Beutler 1930). Similarly, Elisabeth Kleber (1935), who also did her PhD thesis research at von Frisch’s institute, found a positive correlation of bee visits with cyclic pollen production, as well as with daily changes in nectar amount and/or nectar sweetness. At other times, the bees remained in the hive, conserving energy that otherwise would be exhausted on non-productive foraging flights (Kleber 1935). Forty years later, Rainer Koltermann (1971) demonstrated that bees could also be trained to scent and color marking. He was able to shorten the training times to 15 min, which enabled a setting with 9 time points per day and only 45 min inter-training intervals. The bees indeed recalled the correct scent mark every time, which showed that bees can remember up to 9 time points per day. Furthermore, Beling (1929) demonstrated that even one day of training is sufficient to form time memory.^29^ Again, this capability may have co-evolved as an adaptation to short-term flowering.

Nowadays, it is clear that interactions between flowering plants and insect pollinators, such as bees, shape ecological communities. These relations provide one of the best examples of coevolution (for a review see Bloch et al. 2017).

#### Involvement of the circadian clock in foraging and the time memory

The work by several authors has shown that bees continue foraging under constant conditions, with a periodicity slightly different from 24 h (Renner 1955a, 1957; Beier 1968; Beier and Lindauer 1970; Frisch and Aschoff 1987), confirming that the time memory is controlled by an endogenous circadian oscillator. The first demonstration that the bee’s time memory functions independently of external factors was achieved through a spectacular transatlantic-transfer experiment conducted by Max Renner^30^ (1955b, 1957). Two identical flight rooms with constant lighting, temperature, and humidity were built in Munich (even olfactory signals were aligned by letting the same honeybee colony inhabit the flight rooms for several days prior to the experiment) and sent to Paris and New York, where they were reassembled, making sure that they were oriented in exactly the same geographical direction. In collaboration with partnering research institutes in France and the United States, Renner managed to transport bees trained in the flight room in Paris to New York by airplane in only 22 h (including the time for the last training session, customs clearing, and setting up the hive!). Under constant environmental conditions, the bees continued to forage according to their normal approximately 24 h rhythm, regardless of the local time in New York. The reverse experiment, in which freshly trained bees were transferred from New York to Paris, achieved analogous results.

Two years later, in a follow-up experiment, Max Renner addressed the question whether the intrinsically determined pattern of the time sense of honeybees can be influenced by exogenous factors. Bees were trained on Long Island (50 miles northeast of New York City) to collect food at a specific time daily, and then flown overnight to Davis (50 miles northeast of San Francisco) (Renner 1959). The time difference between these two locations is 3 h 15 min. On the first day after translocation, the bees exhibited two maxima in their searching activity: a first peak corresponding to the time of maximum activity on Long Island, and a second peak that occurred 1.5 h later than the first maximum. On the second day, both the first and the second maximum had shifted such that each of them occurred 1.5 h later than the corresponding peaks on the first day. On the third day, the second peak shifted back an additional 30 min, thus corresponding well to the foraging maximum that would have occurred if the bees had been trained to the same local time in Davis as done on Long Island. Renner interpreted the first activity peak as a reflection of the endogenous rhythm of the bee’s time-telling mechanism, whereas he attributed the second activity peak to the effect of exogenous factors, such as the alternation of the day–night rhythm, on this biological clock.

Thus, the endogenous circadian foraging oscillator of the honeybee is entrainable, i.e., it can be synchronized to light–dark (LD) cycles and is able to follow phase-shifts of the LD cycle. Such an adaptation to a phase shift always requires several days (transients), which is typical for circadian clocks. Like other circadian rhythms, the foraging rhythm has a relatively narrow range of entrainment and fails to entrain when LD cycles are shorter than 20 h or longer than 26 h (Beier 1968). The latter result can fully explain the findings of Beling (1929) and Wahl (1932). Taken together, there is no longer any doubt that the time memory of honeybees is based on a circadian clock.

Starting with the classic work by Renner (1957), researchers have investigated how different factors influence the foraging activity profile of time-trained honeybees. Light (Moore and Rankin 1983; Moore et al. 1989), social contact (Medugorac and Lindauer 1967), different weather conditions, as well as the level of experience shape the pattern of foraging activity, which is endogenously controlled by the circadian clock (Moore and Doherty 2009; Moore et al. 2011; Van Nest et al. 2018). Moreover, experimental manipulations like anesthesia (via low temperatures, CO_2_, isofluorine), which phase-shift the circadian clock, affect normal foraging behavior of the bees (Renner 1957, 1959; Medugorac and Lindauer 1967; Cheeseman et al. 2012). Especially anticipatory behavior in foraging rhythms varies with training time length and number of training days. It also depends on weather conditions and experience level of the individual bees, which are all accurately timing their foraging flights (Moore and Doherty 2009; Moore et al. 2011; Moore and Rankin 1983). The role of different foraging strategies and multiple time memories for energetically optimal foraging is discussed in van Nest and Moore (2012), Wagner et al. (2013), and van Nest et al. (2016). A review of a potential linkage between circadian rhythms of locomotor activity and the foraging time sense, as well as a presentation of learning and entrainment hypotheses to explain the mechanism underlying the time sense, can be found in Moore (2001). More recent studies on time memory and clock genes in honeybees have started to address mechanisms of regulation of the honey bee foraging rhythm at the molecular level (Naeger et al. 2011; Jain and Brockmann 2018).

#### Time-compensated sun-compass orientation and communication among bees

In the decades following Beling’s and Wahl’s work, Karl von Frisch decoded the so-called waggle dance of the honeybee, through which the bee communicates the location of a food resource to its nest mates (von Frisch 1940, 1950, 1967). Together with Martin Lindauer he found that bees use the position of the sun and the associated polarized light in the sky as a reference point for orientation. These cues compensate for the daily movement of the sun across the sky with the help of an internal clock, as different time memory experiments with dancing bees have shown (von Frisch und Lindauer 1954, Lindauer 1960; reviewed in Lindauer 1967). Although it seems that the bees prefer landmarks over the sky compass, they use sun-compass orientation when landmarks are missing. Beier and Lindauer (1970) investigated, in a series of experiments, the time memory and sun-compass orientation after shifting the clock by artificial light-cycles in the constant room. Based on the results, they concluded that both consult the same driving mechanism, the endogenous circadian clock. A more recent study demonstrated that bees could integrate circadian time, place, and visual stimuli, thereby forming an episodic-like memory (Pahl et al. 2007). Later studies showed that the bee uses the sun-compass in its initial vector flight and has a cognitive map for the following homing flight (Cheeseman et al. 2014).

An interesting question, also addressed in Karl von Frisch’s laboratory, is how the time-trained bees avoid the recruiting influence of other bees that return from foraging flights at other times of the day and use the waggle dance to encourage the nest bees to follow the announced direction to a food source. Ingeborg Beling (1929) briefly examined this potential issue. As a follow-up of her work, Elisabeth Kleber (1935) noted that time-trained bees hardly ever respond to the waggle dance of other bees foraging outside their trained times. A few years later, Ilse Körner (1940) discovered that an existing time memory reduces the probability of a honeybee being recruited by other foragers. She observed bee behavior in the colony after time training and found that the trained bees withdraw from the comb center and the dancing bees. Only shortly before and during the training time window, they approach the ‘dance floor’ and can be recruited. This indicates that time memory has a high priority when a honeybee makes a foraging decision.

#### Neuronal mechanisms of the time memory

The neuronal mechanisms of the time memory are still elusive. Almost 50 years ago, Lindauer and coworkers transplanted the ‘memory centers’ of insects, the mushroom bodies, of time-trained bees to bees that had not been trained to a certain foraging time. They found that 3 days after the transplantation, the recipient bees foraged at the time to which the donors had been trained. The authors concluded that a humoral transfer of the time signal from the mushroom body of the donor brain to the brain of the recipient is likely (Martin et al. 1978). However, it remained unclear what this humoral substance is and how the circadian clock is connected to the mushroom bodies. One hundred years after Beling’s experiments, we still do not have a definitive answer to these questions, but the recent decoding of the clock neuronal network has shown that at least the circadian clock neurons and their neurites are located in close proximity to the mushroom bodies (Fuchikawa et al. 2017; Beer et al. 2018). This discovery makes it possible for the circadian clock to tell the mushroom bodies the time of the day. Future studies will have to verify this idea.

## Concluding remarks

Beling was an exceptionally gifted scientist and a pioneer in chronobiology. Her precise scientific observations uncovered basic principles of the time memory in honeybees, which subsequently enabled scientists to build on her work—even decades later. Unfortunately, after a few years of extremely productive research, Beling ended her scientific career, probably due to the then enormous political pressure on married women to prioritize family over their careers. Few others, like Ruth Beutler (mentioned above) stayed in academia but were most of the time employed in positions not adequate to their academic qualifications (see the article by Zupanc 2023 in this issue).

Nowadays, policies promote gender equality at universities and, in general, women are no longer underrepresented among students and staff in most academic areas. However, the situation is different in leadership positions: In Germany, only one in four to five professorships is held by a woman, and only around 20% of university rectors/presidents are female.^31^ Nevertheless, we note an improvement, while we hope that talented scientists will be given the opportunity to remain in academia in future, regardless of their gender.

The field of time memory still harbors many aspects that we cannot yet explain. For example, we are only at the beginning of understanding the connection between the circadian clock and the bees’ extraordinary memory for time. Future studies will hopefully continue the work that Ingeborg Beling began almost 100 years ago.

## Data Availability

Data sharing not applicable to this article as no datasets were generated or analyzed during the current study.

## Abbreviations

Barch: Bundesarchiv Berlin-Lichterfelde
BSB: Bayerische Staatsbibliothek
LD cycle: Light–dark cycle
UAM: Universitätsarchiv München

## Footnotes

1 Karl von Frisch (1886–1982) was an Austrian zoologist who received a Nobel Prize in Physiology or Medicine in 1973 for his pioneering work at the interface of ethology and sensory physiology. He is best known as the discoverer of the dance language in bees. He was also co-founder of the *Zeitschrift für vergleichende Physiologie* (the predecessor of the Journal of Comparative Physiology A) and served as its Editor-in-Chief from 1924 to 1960.

2 UAM OC-Npr-1928–29.

3 https://de.wikipedia.org/wiki/Ernst_von_Beling.

4 Position title of the chief executive of many universities in Europe and other countries, comparable to that of president at U.S.-American universities and of vice-chancellor at universities of Commonwealth nations.

5 Municipal Girls’ School at Luisen Street. Emphasis of this school’s curriculum was on modern languages, mathematics, and sciences.

6 The *Abitur* is a qualification awarded upon successful completion of secondary education. It is required for admission to German universities.

7 The biographical information about Beling’s years in Munich is based on the records preserved in the archives of the University of Munich (UAM OC-Npr-1928–29).

8 During the first half of the twentieth century, it was not uncommon to complete the PhD dissertation at a German university within one year and the entire undergraduate/graduate education within five years.

9 In 1976, when Karl von Frisch celebrated his 90th birthday, former students, colleagues, friends, and relatives were invited to reflect, in form of an essay, on memorable interactions with him. Ingeborg Beling contributed a piece to this collection, which includes a vivid recollection of von Frisch’s support of her research work (BSB Ana 540.C.V. 7c).

10 It is no accident that Karl von Frisch recommends the writings of Gottfried Keller. His mother, Marie von Frisch (née Exner) and her brother Adolf Exner were friends with the famous Swiss writer and politician Gottfried Keller (1819–1890). The extensive exchange of letters between Keller and the two siblings is well known in the literary world. Karl von Frisch wrote the foreword to the second edition of the collection of this correspondence (Schmidt I (ed) *Aus Gottfried Kellers glücklicher Zeit. Der Dichter im Briefwechsel mit Marie und Adolf Exner. Vorwort Karl von Frisch*, Stäfa, Zürich (1981)).

11 UAM OC-Npr-1928–29.

12 Ludwig Armbruster (1886–1973) was a catholic priest and zoologist who specialized in the study of bees. He was director of the *Institut für Bienenkunde* and full professor at the Agricultural University of Berlin. In 1934, he was forced to retire from his academic positions at the university, at least in part because of anti-Nazi remarks he had made and of his support of, and collaboration with, Jews.

13 The film was produced in collaboration with the *Verlag für wissenschaftliche Filme* in Berlin. According to Beling (1931b), this publishing company of scientific films went bankrupt. It is unknown whether copies of the film still exist.

14 The biographical information about Ingeborg Beling during her tenure at the Biologische Reichsanstalt is based on two sources: personal notes that she wrote around 1985 when she visited her daughter Ingrid in Lima (Peru); and a biographical sketch of Ingeborg Beling in Koronowski C (1998) Vom Gartenmädchen Balloniak bis zur Abteilungsleiterin Dr. Erika von Winning: Frauen in den Anfängen der Forschungsanstalt, ihre Ausbildung, Anstellungsbedingungen und heutige Situation, ergänzt durch Kurzbiographien von Frauen mit 25jähriger und längerer Tätigkeit in der Forschungsanstalt. Mitteilungen aus der Biologischen Bundesanstalt für Land- und Forstwirtschaft, Issue 336. Parey Buchverlag, Berlin. Our attempts to retrieve more information about the ‘Berlin years’ have been unsuccessful. Neither at the *Bundesarchiv* (that has in its possession some of the personnel files of the *Biologische Reichsanstalt*) nor at the Julius Kühn-Institut (today’s successor of the *Biologische Reichsanstalt*) any files related to Ingeborg Beling could be found. It is possible that her personnel file was destroyed in the course of several reorganizations of the *Biologische Reichsanstalt* and its successors since the end of World War II.

15 Kater, M.H. (1972) Krisis des Frauenstudiums in der Weimarer Republik. VSWG 59, 207–255.

16 Arndt Michael Albrecht Hase (1882–1962) was a German entomologist and parasitologist. He played a leading role in the application of poison gas for pest control. In collaboration with the toxicologist Ferdinand Flury (1877–1947), he was involved in the development of Zyklon A in the early 1920s. This hydrogen cyanide-based pesticide was used for fumigation of soldiers’ barracks and prison camps. Zyklon A was further developed by German chemists into Zyklon B, which became a major means for the mass murder of Jews in the gas chambers of concentration camps during World War II.

17 After her marriage to Rudolf Freiherr von Stein zu Nord- und Ostheim, Ingeborg Beling changed her name to ‘Ingeborg Freifrau von Stein-Beling’ and then published under her new author’s name, ‘Ingeborg von Stein-Beling.’ In her essay written when Karl von Frisch celebrated his 90th birthday, she also included the *Freifrau* (Baroness) title of nobility in her name.

18 Ingeborg von Stein-Beling had three children, Eckart (1934–2000), Dietrich (born 1937), and Ingrid (1942).

19 Such policies were implemented soon after the appointment of Adolf Hitler as chancellor on January 30, 1933. The reason why only women, but not men, were targeted by these policies was clearly articulated in the first public speech that Joseph Goebbels (1897-1945) gave as Minister for Public Enlightenment and Propaganda on March 18, 1933, when he emphasized that “the highest, optimal, and most adequate priority for women should be in the family, and the most wonderful task they can fulfill is to bear children to their country and *Volk.*” (https://archive.org/details/193303XxJosephGoebbelsEroeffnungsredeEinerAusstellungInBerlin49s).

20 A scan of this letter was provided by Ingrid Bettin-Heitmann, the daughter of Ingeborg von Stein-Beling, to GKHZ on November 7, 2023.

21 Interview through video conferencing of Ingrid Bettin-Heitmann by GKHZ on October 2, 2023.

22 BSB Ana 540.C.V. 7c.

23 A digitized version of this film, stored in the audiovisual archive of the Österreichische Mediathek, in association with the Technical Museum Vienna, is available at the following link: https://www.mediathek.at/katalogsuche/suche/detail/?pool=BWEB&uid=018AA01B-256-01815-00000484-0189A3E5&cHash=6084cbdafdc52f3350fcd5b037f414cf. According to the information provided on the website, this scientific film was produced in 1949. However, since the publication date is not indicated, it is possible that some shots for this film were taken at different times, including the summer of 1952, when according to the recollection of Ingrid Bettin-Heitmann, the scenes showing her mother were taken.

24 Available, for example, on the video-sharing technology platform Dailymotion: https://www.dailymotion.com/video/x8gq9td.

25 Information provided by Ingrid Raab of the Meldebehörde of Kleinwallstadt, via email to GKHZ on August 3, 2023, and November 6, 2023.

26 Oskar Wahl was born on December 29, 1906. Like Ingeborg Beling, he did his PhD thesis research under Karl von Frisch at the Zoological Institute of the University of Munich. He published his thesis in 1932 under the title *Neuere Untersuchungen über das Zeitgedächtnis der Bienen* (Further investigations on the time memory of bees). Wahl remained at the Institute for several years. Starting in 1935, he searched for a denaturant for bee sugar, a research project funded through a grant awarded by the German Research Foundation to Karl von Frisch (BArch, R 73/15459). Later he was affiliated with the University of Marburg and headed the Bee Institute in Kirchhain (Hessen). Oskar Wahl carried out fundamental research into the protein (pollen) nutrition of bee colonies. From 1970 to 1979, he was the German editor of *Apidologie*. After his retirement, he worked for seven years at the Institute of Apiculture, Lunz am See (Austria). There, he focused on the influence of protein nutrition of bees in the context of their sensitivity to pesticides.

27 Many plants only open their flowers at certain intervals during the day to save pollen and optimize fertilization. Carolus Linnaeus (also known as Carl von Linné after his ennoblement in 1761) observed this regular opening and closing of flowers in his garden and set up a flower clock to indicate these times. He claimed that he didn’t need a watch, as he only had to check which species in his garden had opened or closed their flowers at a given time to figure out the time of day.

28 Some other researchers, like Cremer (1923), suspected an unknown external factor that organisms on Earth use as a clock because they failed to observe oscillating activity of organisms after removing all cyclic changes in environmental conditions. Interestingly, although Cremer (1923) could not observe continuous cyclic movement of plant leaves under constant conditions in a salt mine, Wahl (1932) observed continuous cyclic foraging activity of bees in the same salt mine.

29 More recent experiments by Moore and Doherty (2009) have shown that some foraging honeybees can remember the feeding time even if just a single visit to a feeding station is rewarded.

30 Maximilian ‘Max’ Renner (1919–1990) was a German zoologist who worked at the Zoological Institute of the University of Munich — first as a scientific assistant under Karl von Frisch, then as an adjunct professor heading his own research group. He is best known for his transatlantic transfer experiment which was a milestone in the establishment of the time memory of honeybees as an intrinsic time-keeping mechanism.

31 CHE report U-Multirank Gender Monitor 2022; www.umultirank.org/export/sites/default/.galleries/generic-images/Gender-Monitor-2022/u-multirank-gender-monitor-2022.pdf.
